# The Environmental Impact of Medical Imaging Agents and the Roadmap to Sustainable Medical Imaging

**DOI:** 10.1002/advs.202404411

**Published:** 2025-02-04

**Authors:** Verena Pichler, Ricardo P. Martinho, Lisanne Temming, Tim Segers, Frederik R. Wurm, Olga Koshkina

**Affiliations:** ^1^ Department of Pharmaceutical Sciences Division of Pharmaceutical Chemistry University of Vienna Vienna 1090 Austria; ^2^ Biomolecular Nanotechnology Group Department of Molecules and Materials MESA+ Institute for Nanotechnology Faculty of Science and Technology University of Twente Enschede 7522 The Netherlands; ^3^ Sustainable Polymer Chemistry Department of Molecules and Materials MESA+ Institute for Nanotechnology Faculty of Science and Technology University of Twente Enschede 7522 The Netherlands; ^4^ BIOS / Lab on a Chip Group Max Planck Center Twente for Complex Fluid Dynamics MESA+ Institute for Nanotechnology University of Twente Enschede 7514DM The Netherlands; ^5^ Phos4nova B.V. Enschede The Netherlands

**Keywords:** computed tomography, contrast agents, magnetic resonance imaging, nuclear imaging, ultrasound imaging

## Abstract

Medical imaging agents, i.e., contrast agents for magnetic resonance imaging (MRI) and radiopharmaceuticals, play a vital role in the diagnosis of diseases. Yet, they mostly contain harmful and non‐biodegradable substances, such as per‐ and polyfluoroalkyl substances (PFAS), heavy metals or radionuclides. As a result of their increasing clinical use, these agents are entering various water bodies and soil, posing risks to environment and human health. Here, the environmental effects of the application of imaging agents are outlined for the major imaging modalities, and the respective chemistry of the contrast agents with environmental implications is linked. Recommendations are introduced for the design and application of contrast agents: the 3Cs of imaging agents: control, change, and combine; and recent approaches for more sustainable imaging strategies are highlighted. This combination of measures should engage an open discussion, inspire solutions to reduce pollution by imaging agents, and increase awareness for the impact of toxic waste related to imaging agents.

## Graphical abstract

Medical imaging agents are vital for diagnosis, but their harmful components pollute water and endanger health. This article examines their environmental impact, linking their chemistry to consequences. It proposes the 3Cs (control, change, combine) as guidelines for sustainable design, and highlights innovative, eco‐friendly imaging approaches.

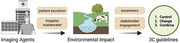

## 1 Introduction

**Figure 1:**
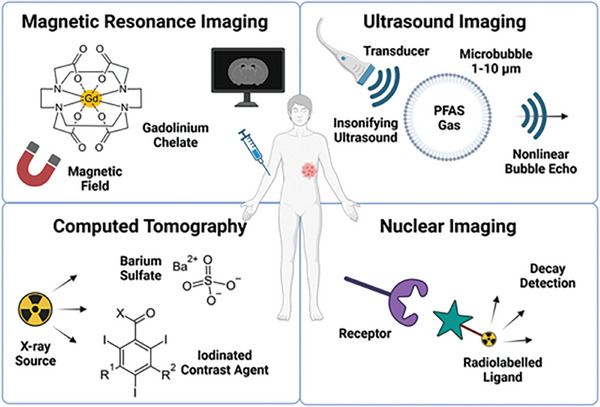
Representative examples of clinically used imaging agents. Gd‐chelates or superparamagnetic iron‐oxide nanoparticles are used in MRI. Contrast‐enhanced ultrasound imaging uses gas‐filled microbubbles to generate acoustic contrast. Common CT agents are based on barium sulfate or iodinated compounds, which increase absorption of X‐rays. Nuclear imaging uses radioactive tracers that target specific cells and emit either positrons (PET) or gamma rays (SPECT) upon decay, which is detected by specialized cameras.

Medical imaging plays a crucial role in the diagnosis, monitoring, and treatment of clinically‐relevant diseases but also in our understanding of physiological processes.^[^ ^1^ ^]^ Detailed images of organs, tissues, and bones enable precise identification of diseased tissues or altered biochemical functions, necessary for setting up effective treatment plans.^[^ ^2^ ^]^ Currently, the most spread techniques in the clinic are magnetic resonance imaging (MRI), ultrasound (US), X‐ray‐computed tomography (CT), and nuclear medicine imaging, e.g. positron emission tomography (PET) and single‐photon emission computed tomography (SPECT) (**Figure** **1**). These imaging techniques benefit from the injection of medical imaging agents, also called contrast agents, radiotracers, and sometimes probes, depending on the imaging technique and their mode of action. Imaging agents highlight regions of interest, including tumors, inflammation, and vascular obstructions. Earlier reports estimated that there are over 80 million usages of contrast agents per year,^[^ ^3^ ^]^ which corresponds to over 8 million liters of contrast media usage.^[^ ^4^ ^]^ The market size is over USD 5.5 billion per year across all modalities, with the expectation of significant growth in the coming years.^[^ ^5^ ^]^ Each of these agents can have certain implications on health and environment, depending on chemical composition, that are discussed in this perspective.

Imaging agents typically contain harmful substances such as heavy metals, radionuclides or per‐ and polyfluoroalkyl substances (PFAS).^[^ ^6^ ^]^ As a result, imaging agents pose environmental risks in the whole value chain: The production of imaging agents relies on non‐renewable resources, requires mining, energy‐intense reformulation of heavy metals, and toxic process chemicals. More importantly, the end‐of‐life of imaging agents needs to be considered: imaging agents must be chemically stable to omit unwanted reactions and degradation inside of the human body, for example, to prevent the release of free and potentially toxic metal ions. Their high stability and persistence can lead to accumulation either inside of the body or after excretion from the patient in the environment, which can lead to adverse effects on flora and fauna, including humans.^[^ ^7^, ^8^ ^]^

This perspective article aims to initiate the discussion about considering the end‐of‐life of medical imaging agents, to initiate solution‐oriented thinking and foster research on more sustainable or environmentally benign imaging alternatives. We aim to raise awareness of environmental accumulation of heavy metals and other “forever chemicals” within groups of different stakeholders, including clinicians, researchers within the imaging community, and industry and lead to creation of ideas for potential solutions. We focus on medical imaging agents used in the major clinical imaging techniques, such as MRI, ultrasound, X‐ray‐CT, and nuclear medicine imaging. Throughout this perspective, we use imaging agents as a general term, and specific terms, such as contrast media or radiotracers in respective sections to use the nomenclature adapted by a particular community. We outline current chemistry and applications of imaging agents, and subsequently discuss the sustainability concerns and show the few more sustainable alternatives which are reported to date. Finally, we propose a guideline for future design of medical imaging agents to minimize the environmental impact introducing the 3C principle: control, change, and combine.

## 2 Overview of Clinical and Commercially Available Imaging Agents: Chemistry and Applications

To discern the implications of employing imaging agents, we provide an overview of the prevalent imaging agents utilized in clinical settings, whether commercially available or produced in‐house. The environmental footprint of imaging agents varies significantly depending on the modality employed, underscoring the complexity in formulating comprehensive guidelines for designing sustainable imaging agents given the potentially conflicting needs, requirements, and challenges. **Table** **1** presents a summary of the challenges identified within each modality.

**Table 1:** Examples of current imaging agents and their potential concerns.

Modality	Imaging agent classification	Potential concerns
MRI	^19^F agents PFC/PFAS (currently some are in clinical trials; commercial clinical grade agents)	Classification as environmental pollutant/”forever chemical”
	Gd^3+^ chelates (^1^H MRI / T_1_; the most used in the clinic)	Accumulation in brain and tissue Water pollution and potential release of toxic Gd^3+^
	SPIO (^1^H MRI/ T_2_; clinical approval in some courtiers; few formulations in clinical trials, some retracted)	Aggregation, toxicity due to Fe‐ion release
	Mn^2+^ chelates (^1^H MRI/ T_1_; clinically approved or in clinical trials)	Environmental pollutant and associated with human health effects of Mn^2+^
US	PFC/PFAS (clinically approved)	Classification as environmental pollutant/”forever chemical”
	SF_6_ (clinically approved)	Greenhouse gas
CT	ICM (clinically approved)	Accumulation in aquatic bodies Reaction with disinfection reagents creating toxic chemicals
PET/SPECT	Production of radionuclides	End disposal of outdated reactors
	Decay products of harm	Technetium‐99, Yttrium‐89 or Hafnium‐177 accumulation in water bodies
	Production of radiotracers	High amount of plastic was labeled as radioactive waste

### 2.1 Magnetic Resonance Imaging (MRI)

MRI uses a strong magnetic field and non‐ionizing radiofrequency waves to generate images based on nuclear magnetic resonance, primarily detecting the water and fat distribution in tissues (we recommend references^[^ ^9^, ^10^, ^11^ ^]^ for details on MRI). The MRI signal intensity is influenced by the molecular environment and mobility, impacting longitudinal (*T*_1_) and transverse (*T*_2_) MR relaxation times, i.e., the times required to reestablish thermal equilibrium after the application of a radiofrequency pulse. ^1^H MRI contrast agents alter the relaxation times of protons, increasing or reducing the MRI signal intensity.^[^ ^6^, ^12^, ^13^ ^]^

Paramagnetic gadolinium (Gd^3+^) chelates, or gadolinium‐based contrast media (GBCA) are today the most used clinical ^1^H MRI contrast agents for *T*_1_‐weighted contrast. They are usually injected intravascularly.^[^ ^6^, ^13^ ^]^ Gadolinium ions are toxic as they interfere with Ca^2+^ pathways but chelation leads to stable Gd‐complexes that are considered to be safe.^[^ ^14^ ^]^ The accumulation of Gd in brain and tissues, however, raised the discussion in the MRI field outlining the need to reduce the Gd‐containing imaging agents.^[^ ^15^, ^16^, ^17^, ^18^, ^19^ ^]^ To reduce the toxicity, Gd‐chelates with further improved stability were developed, switching from linear to macrocyclic chelators.^[^ ^15^, ^16^, ^17^, ^18^, ^19^ ^]^

Besides gadolinium, manganese‐based contrast agents have been developed,^[^ ^20^, ^21^ ^]^ which also induce *T*_1_‐weighted contrast. Mangafodipir (MnDPDP) inclusively has an approval by the Federal Drug and Food Agency (FDA) for liver imaging.^[^ ^22^ ^]^ Additionally, superparamagnetic iron oxide nanoparticles (SPIO), which typically produce *T*_2_‐weighed contrast, have been explored.^[^ ^23^ ^]^ SPIO are considered as non‐toxic but can suffer from aggregation, unwanted organ accumulation, and iron‐associated side effects.^[^ ^24^, ^25^, ^26^, ^27^, ^28^ ^]^ After the initial approval of several formulations, recently the use of SPIO decreased as the FDA retracted all initially approved SPIO. Nevertheless, a few promising SPIO are currently undergoing clinical trials.^[^ ^29^, ^30^, ^31^, ^32^, ^33^ ^]^

In addition to ^1^H MRI, in the last two decades, heteronuclear—so‐called “hotspot”—MRI has shown great promise. Instead of varying the contrasts as done in in ^1^H MRI, heteronuclear MRI detects other MR‐active nuclei such as ^23^Na, ^31^P, or ^19^F directly, in a different channel than protons as a new “color”.^[^ ^34^, ^35^, ^36^ ^]^ So far, only ^19^F MRI agents are commercially available and used in clinical trials. ^19^F MRI is a background‐free technique, allowing quantification, and allows for application in tracking immune cell therapies and inflammation imaging.^[^ ^36^, ^37^, ^38^, ^39^, ^40^, ^41^, ^42^ ^] 19^F MRI contrast agents consist of formulations of liquid PFAS also referred to as perfluorocarbons (PFC). However, the high chemical stability of the C─F bond in PFC/PFAS‐based agents are non‐degradable, resulting in their categorization as environmental pollutants and “forever chemicals.”^[^ ^43^, ^44^ ^]^

### 2.2 Ultrasound Imaging

Ultrasound contrast agents (UCAs) comprise a suspension of gas‐filled microbubbles with diameters ranging from ca. 1 to 10 µm.^[^ ^45^ ^]^ As their size is smaller or similar to that of red blood cells, UCAs can pass through even the smallest capillary blood vessels after intravenous injection. UCA microbubbles resonate to ultrasound frequencies used in medical ultrasound imaging (1–10 MHz) through which they produce a strong echo that can be 1 billion times stronger than that of red blood cells allowing the visualization and quantification of organ perfusion. Typical applications of UCAs include the evaluation of renal lesions,^[^ ^46^ ^]^ liver metastases,^[^ ^47^ ^]^ myocardial perfusion,^[^ ^48^ ^]^ and thyroid nodules.^[^ ^49^ ^]^

UCA microbubbles are stabilized against dissolution and coalescence by a shell of phospholipids, proteins, or polymers. To further increase the stability of the bubbles, their gaseous core is filled with a gas with a low solubility in blood. Today, the clinically available UCAs are filled with gaseous PFCs/PFAS as well as sulfur hexafluoride (SF_6_), i.e., with either SF_6_, C_3_F_8_, or C_4_F_10_, providing the bubbles with an in vivo circulation time of ca. 10 min. The inert gas cannot be metabolized and will be exhaled within minutes after disintegration of the bubbles, while the phospholipids from the shell are metabolized by the liver. PFAS are considered as forever chemicals and SF_6_ is known as a greenhouse gas that is 23500 times more potent than CO_2_ (cf. Section 3.2 for discussion of environmental concerns).^[^ ^50^ ^]^

### 2.3 X‐Ray Computed Tomography

Computed tomography (CT) uses X‐rays to create cross‐sectional images of the body, based on a different absorption and attenuation of X‐rays. The interactions of X‐rays with materials generally depend on their density and atomic number.^[^ ^51^ ^]^ Thus, CT contrast agents contain heavy elements, such as iodine or barium.

Iodinated contrast media (ICM) contain inert, i.e., non‐biodegradable, triiodobenzene derivates, which are not metabolized and excreted though the kidney (Figure 1).^[^ ^52^ ^]^ Iodinated contrast agents are used, for example, for functional assessment of the blood‐brain barrier^[^ ^53^ ^]^ and perfusion CT.^[^ ^54^ ^]^ Barium‐containing contrast agents are used for upper gastrointestinal examinations and for identifying anatomic lesions in the small bowel, colon, or rectum.^[^ ^55^, ^56^ ^]^

Due to the need for safe CT contrast agents, the stability and therefore persistence of ICM was significantly improved in recent years. Being halogenated compounds, excreted stable ICMs are long lasting pollutants. Additionally, their breakdown products can expose harm to water bodies and end up in drinking water.

### 2.4 Nuclear Imaging—PET, SPECT Imaging, and Theranostics

Nuclear Imaging is the first choice when it comes to functional imaging: either a metabolic process or receptor‐ligand interaction can be visualized to detect, monitor, or clarify biological functions. Dependent on the radionuclide used, the detection either takes place via gamma emission for SPECT or positron emission for PET.^[^ ^57^ ^]^ Each radiotracer needs to be carefully designed and prepared to allow imaging of the respective biological function. The choice of radionuclide is adjusted based on the imaging methodology, the biological half‐life time and the purpose of the biochemical process.^[^ ^58^ ^]^ In general, radionuclides can be separated into two groups: organic^[^ ^59^ ^]^ and inorganic radionuclides.^[^ ^60^ ^]^ The most common radionuclide involved in SPECT is the gamma emitter technetium‐99 m with a half‐life time of 6 hours. The success of technetium‐99 m is mainly based on the affordability and easy access of the radionuclide generators.^[^ ^61^, ^62^ ^]^ Other radionuclides involve iodine‐125 and iodine‐131, which can be applied as a theranostic pair especially in thyroid cancer.^[^ ^63^ ^]^

PET offers a higher resolution, however, demands special equipment such as a medicinal cyclotron and radiopharmaceutical synthesizers. Besides fluorine‐18, also gallium‐68^[^ ^64^ ^]^ was celebrating an enormous success after the technetium‐99 m crisis and the associated shortage of ^99m^Tc‐generators.^[^ ^65^ ^]^

Recently, theranostic concepts were introduced on the pharmaceutical market, with Luthatera^[^ ^66^ ^]^ and Pluvito^[^ ^67^, ^68^ ^]^ receiving the market authorization in 2018 and 2022, respectively. Here, a targeting moiety is labelled with a diagnostic nuclide (radionuclides emitting positron or gamma rays, e.g. gallium‐68) to achieve high quality imaging, the radionuclide is then replaced by a therapeutic one (radionuclides emitting ß^−^ or alpha particles, e.g. lutetium‐177) allowing subsequent radiotherapy.

The need for continuous production and supply of radionuclides and radiotracers requires not only high levels of infrastructure, supply routes and plastic consumables, but also the end disposal of activated radioactive waste has to be considered.

## 3 Impact of Medical Imaging Agents on Health and Environment

The pollution caused by medicinal residues is a growing concern, recognized by the World Health Organization,^[^ ^69^ ^]^ United Nations,^[^ ^70^ ^]^ the European Union, and several national governments.^[^ ^71^, ^72^, ^73^, ^74^ ^]^ In this regard, the environmental hazards of medical imaging agents are discussed within ecology community.^[^ ^75^, ^76^ ^]^ However, there is no or only very little cross‐talk between ecologists and the community developing, and producing imaging agents and product users such as clinicians, making it necessary to have a closer look at different lifecycle stages.

**Figure 2:**
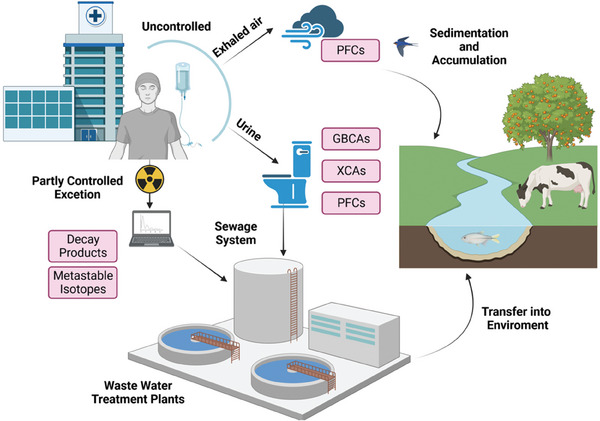
Distribution and routes of imaging agents entering water and soil. After urinary excretion by patients, imaging agents follow the pathway from hospital wastewater to sewage treatment plants (WWTPs) and ultimately to rivers and other natural bodies of water. PFC contrast agents reach water bodies via sewers, while manufacturing processes are the biggest source of PFCs in the air.

Today, medical imaging paves the way toward personalized medicine and the increased clinical use allows harmful and non‐biodegradable substances contained in imaging agents to enter the environment (**Figure** **2**). To estimate the impact of imaging agents’ accumulation on human health, longer‐term studies are still missing.^[^ ^77^, ^78^ ^]^ It furthermore depends on the removal efficiency of contrast agent from wastewater by wastewater treatment plants (WWTPs). Currently, the removal of various medical imaging agents by WWTPs is insufficient. Moreover, the high stability and results in environmental accumulation. However, changes in the milieu, such as transfer to sea water, temperature change UV irradiation etc. can lead to degradation of the imaging agent. So far also here only very little data is available. Therefore, a precise monitoring of agents’ concentration in the environment is essential to minimize the health risks.

The generation of potentially harmful components can occur throughout the whole lifecycle of imaging agents, including the manufacturing process, application to patients, and their subsequent metabolism leading to excretion. In general, contrast agents are usually designed to be highly stable and not metabolized, thus excluding unwanted pharmacological effects. Imaging agents enter the environment primarily as liquid and solid forms (at ambient temperature) based on small molecules, like MRI and CT‐contrast agents, which are introduced into the wastewater system through the patients’ urine (Figure 2). Gaseous ultrasound contrast agents are exhaled directly into the atmosphere (Figure 2). In the following parts of this section, we discuss the implications of each contrast agent type, highlighting the need for future developments of sustainable, environmentally benign contrast agents.

Initial studies have already described the accumulation of imaging agents over time in the air, water bodies, and sediment. However, significantly more research is needed to enable a thorough safety assessment. The evaluation of the downstream impact on human health from the unintentional intake of these environmental residues is hindered by limited data and requires more critical discussion.

### 3.1 Gadolinium‐Based MRI Contrast Agents

Gd‐based MRI agents are the main source of anthropocentric gadolinium contamination in waters. Each year, tens of millions of MRI exams are performed worldwide, resulting in Gd‐use of at least 40000 kg,^[^ ^79^ ^]^ along with additional chemicals needed during the synthesis of chelates and final formulations. Due to their high stability, Gd‐chelates persist in the environment for extended periods, leading to accumulation in waters, such as rivers, lakes,^[^ ^80^, ^81^ ^]^ and coastal waters.^[^ ^76^, ^82^ ^]^ After being discharged in the sewage, Gd^3+^ chelates enter WWTPs.^[^ ^83^ ^]^ However, most WWTPs are not efficient at removing GBCAs causing Gd‐chelates re‐enter the environment as effluent.^[^ ^84^ ^]^ Only a small fraction, roughly 10%, of the total Gd is eliminated during treatment processes.^[^ ^85^ ^]^ Additionally, there is a concern regarding the degradation and dissociation of chelated GBCAs to release free Gd^3+^‐ions during wastewater treatment.^[^ ^86^, ^87^ ^]^ The insufficient retention of Gd‐chelates by WWTPs can induce Gd accumulation in aquatic flora and fauna. Positive Gd anomalies have been detected in shells and soft tissues of various bivalves mollusk shells, for example in shells of great scallops, and soft tissues of dog cockles, in both fresh and seawater.^[^ ^88^, ^89^, ^90^ ^]^ While often no adverse effects on animals were observed, the mussel species *Mytilus galloprovincialis*, exposure to Gd showed decreased metabolic activity and caused oxidative stress and neurotoxicity.^[^ ^91^ ^]^ So far, there are indications that the type of contrast agent, e.g., older generation linear compounds versus chelates, can affect the bioaccumulation and its persistency. Further studies are required to establish a link between the structural properties and bioaccumulation, and long‐term dissociation of Gd from chelates.

Overall, GBCAs’ pollution of water bodies poses potential risks on aquatic life, and there is a possibility that GBCAs could enter the human food chain through contaminated animals, subsequently posing a risk to human health. Also, the unintentional consumption of toxic Gd^3+^ through tap water, posing a risk to human health. WWTPs' inability to effectively remove GBCAs has resulted in elevated levels of anthropogenic Gd in surface and groundwater worldwide, including urban areas reported for several cities, such as Tokyo,^[^ ^92^ ^]^ Wuhan,^[^ ^83^ ^]^ San Francisco,^[^ ^93^ ^]^ and Berlin.^[^ ^94^ ^]^ Gd^3+^ was detected in the municipal tap water of Berlin, and other German cities^[^ ^95^ ^]^ and in the drinking water of Prague^[^ ^96^ ^]^ and London.^[^ ^97^ ^]^ Alarmingly, Gd^3+^ was found in a tap water‐based soft drink (Coca‐Cola) being sold at fast food restaurants.^[^ ^95^ ^]^ Thus, GBCAs are entering the human food chain through tap water, leading to widespread contamination in multiple European cities, and posing public health concerns.

### 3.2 Ultrasound and MRI Agents Based on PFAS

PFAS/PFC are used in both ^19^F MRI (liquid PFAS) and ultrasound (liquid and gaseous PFAS). However, PFAS are concerning due to their extremely high stability, which raised public discussion on their use and led to the ban of several PFAS: PFAS are non‐degradable, so‐called “forever chemicals” with high persistence in the environment, meaning that even if the PFAS pollution by contrast agents is small compared to other applications it can lead to PFAS accumulation in the long‐term. For example, the atmospheric lifetime of SF_6_ used in UCAs is 3200 years.^[^ ^50^ ^]^ In the EU and the US, there is a current discussion to ban all perfluorinated compounds.^[^ ^98^ ^]^

PFAS are a large group of fluorinated chemicals used in a broad range of applications, e.g., textiles, lubricants, food packaging.^[^ ^99^, ^100^, ^101^ ^]^ However, PFAS have been linked to numerous environmental and health concerns.^[^ ^43^, ^102^, ^103^ ^]^ Prolonged exposure to PFAS‐based surfactants, e.g., perfluorooctanoic acid (PFOA) and perfluorooctanesulfonic acid (PFOS) was linked to various disorders, including a weakened immune system,^[^ ^104^ ^]^ increased cholesterol levels,^[^ ^105^ ^]^ thyroid disorders,^[^ ^106^ ^]^ and increased risk of cancer.^[^ ^107^ ^]^ PFAS exposure typically occurs through contaminated water and food.^[^ ^108^ ^]^ PFAS have been reported in various water sources,^[^ ^109^ ^]^ including groundwater,^[^ ^110^, ^111^ ^]^ surface waters (i.e., rivers and lakes),^[^ ^102^, ^112^ ^]^ and drinking water.^[^ ^113^, ^114^ ^]^

The role of imaging in the environmental release of PFAS is small compared other applications, yet concerning due to their high persistence. PFAS that originate from ultrasound agents are excreted from the body, primarily through exhalation. This amounts to only a few microliters per dose. Most PFAS gas volume is contained in the headspace of the vials in which the contrast agent is stored. Additionally, during the production process of some UCAs, the entire production machinery is filled with the bubble filling gas further increasing the amount of PFAS gas released in the atmosphere. Admittedly, PFCs are among the most potent and long‐lasting types of greenhouse gases emitted by human activity.^[^ ^115^ ^]^ The route of liquid high boiling point PFAS used in ^19^F MRI such as perfluorooctyl bromide and perfluoro‐15‐crown‐5 ether is not completely clear thus far. It was proposed that such PFAS are also removed through exhalation, yet so far there is no direct proof of PFAS in the exhaled air. As ^19^F MRI is in clinical trials today, no data on environmental pollution caused by ^19^F MRI agents has been reported so far. However, in light of the presence of the currently used Gd‐based MRI contrast agents in the environment, it is expected that clinical use of ^19^F MRI would lead also to PFAS pollution. Considering the high persistence and environmental risks, the use of PFAS contrast agents requires very careful consideration, as in the long term even small amounts can lead to accumulation in the environment.

PFAS are considered to impact human health in different aspects. Recent studies describe the impact of PFAS on the thyroid hormone system, female reproductive health,^[^ ^116^, ^117^ ^]^ and potential carcinogenic mechanisms, among others.^[^ ^118^ ^]^

### 3.3 CT‐Agents: Barium Sulfate and Iodinated Contrast Media

Approximately 300 000 000 CT scans are performed per year worldwide with ca. 40% of them being contrast‐enhanced.^[^ ^119^ ^]^ Iodinated contrast agents are the most commonly employed, followed by barium sulfate. Iodinated contrast media (ICM) are administered in doses of 100–200 g per adult patient,^[^ ^120^ ^]^ resulting in ca. 500 tons of waste from ICM per year in Germany alone.^[^ ^121^ ^]^ While the release of barium sulfate is considered unproblematic,^[^ ^122^ ^]^ the persistence of iodinated contrast agents in the environment is seen as a growing problem.^[^ ^52^ ^]^

As ICM are not completely removed in WWTPs, they have been detected in different waters.^[^ ^123^ ^]^ High concentrations of ICM were found in large rivers, such as the Danube and the Rhine. ICMs were also detected in tap water, in Madrid and the Jiangsu province, China.^[^ ^52^, ^75^, ^124^ ^]^ ICMs were also found in aquatic fauna, including bivalves mollusks,^[^ ^8^ ^]^ and in the brain and gonads of fish.^[^ ^125^ ^]^ Different to iodinated agents, barium sulfate is reported as safe to use, as it has very low water‐solubility and belongs to natural minerals with limited adverse effects on the environment. Even though Ba^2+^ ions and soluble salts (particularly BaCl_2_, Ba(OH)_2_) are considered as toxic,^[^ ^126^ ^]^ Ba^2+^ usually precipitates in waters to insoluble salts.^[^ ^127^ ^]^

Humans are mainly exposed to ICM through drinking water^[^ ^128^ ^]^ but little is known about the consequences of ICM‐contaminated water on human health. There is increasing evidence that some of their degradation products are toxic.^[^ ^129^ ^]^ In drinking water, ICM can react with commonly used disinfectants during water treatment, such as chlorine. In the presence of organic material, so‐called iodine‐containing disinfection by‐products can be formed. Compared to by‐products containing chlorine and bromine, iodinated were reported to be more toxic.^[^ ^129^, ^130^ ^]^

### 3.4 Radiotracers and Their Decay Products

Nuclear medicine was long considered to have minimal impact on radioactive waste production due to the use of radionuclides with short half‐lives and their application in small doses. Until recently, radiopharmaceuticals were a niche applied and produced exclusively in hospitals. With the success of theranostic applications and the involvement of the pharmaceutical industry, the situation has changed rapidly.

Radionuclides are produced either in nuclear reactors or medicinal cyclotrons.^[^ ^131^ ^]^ Now, the radiopharmaceutical community is facing the problem of outdated and aged nuclear reactors, while still having to deal with the end‐disposal. Generators allow a more efficient distribution and usage of certain reactor‐based radionuclides, whereas the spend radionuclide generators offer the possibility of reusage and recycling^[^ ^132^ ^]^ Medicinal cyclotrons are now increasingly installed allowing independence from delivery chains and low radioactive waste production within their lifetime. During decommission the main environmental hazard is based on the activation problem of the surrounding material.^[^ ^133^ ^]^

Organic radionuclides can be considered to be the greener alternative to heavy metals in terms of decay products, with fluorine‐18 decaying to oxygen‐18 and carbon‐11 to boron‐11. However, organic radiochemistry requires inert and harsher conditions along with the usage of organic solvents being considered harmful to the environment, like DMF and THF. On the opposite, radiometals are chelated to the respective biomarker in aqueous solutions which can be considered green chemistry.^[^ ^134^, ^135^ ^]^The fate of potentially harmful decay products has received little attention in literature, except for Technetium‐99m.^[^ ^136^ ^]^ For instance, the increased release of Yttrium‐89^[^ ^137^ ^]^ as a stable decay product of Zirconium‐89 may raise concerns.

One further aspect of radiopharmaceuticals includes the continuous production of the radiopharmaceuticals on a daily base due to their short half‐lives. Radiopharmaceutical production under GMP regulation is built‐up on single‐use plastic‐based cassettes, which end up in large amounts of radioactive solid waste in hospitals.^[^ ^138^ ^]^ Depending on a country's radiation safety regulations, the plastics may end up in waste incineration systems, with limited opportunities for recycling.

Nuclear Imaging production is already a highly regulated field, as the administration of radioactivity has to follow the radiation safety regulations.^[^ ^139^ ^]^ This involves the monitored and controlled release of radioactive wastewater from hospitals' sewage systems. Thereby large water tanks and decay systems have to be installed within the hospital.^[^ ^140^, ^141^, ^142^ ^]^ Aqueous medicinal waste has to be stored until the radioactivity level drops below the clearance level, subsequently, the water can be released in the municipal wastewater system. The current medicinal radioactive waste processing and monitoring, however, does not involve the previously mentioned long‐lived or stable decay products Technitium‐99, Yttrium‐98, Hafnium‐177, or others.

Technetium‐99 m is the working horse of nuclear medicine and its decay product ^99^Tc was found in waters^[^ ^143^, ^144^ ^]^ and the soil.^[^ ^145^ ^]^ Also, the success of the theranostic concept comes at a price, the outpatient policy will lead to an increased and uncontrolled excretion of radiometals and their decay products into the environment. The globalized world allows the application of radiopharmaceuticals in developed and equipped hospitals with subsequent patient travel back to their home destinations. For outpatient treatments, excretion can happen in countries different from the point of administration without controlled hospital sewage systems.^[^ ^146^ ^]^ Safety risks of outpatient travel were observed at airports, where the still radioactive patients were  identified during security checks.^[^ ^147^ ^]^

**Figure 3:**
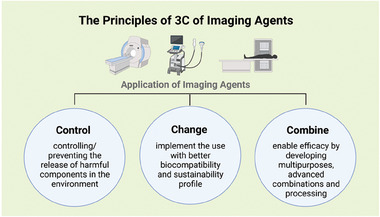
Proposed 3C guidelines for the handling and design and of medical imaging agents to reduce the pollution and facilitate ecologically friendly solutions.

The medicinal radionuclide and radiopharmaceutical market is expected to grow by approx. 4–5% CAGR,^[^ ^148^, ^149^ ^]^ the theranostics market will expand even more drastically by an expected approx. 10% CAGR^[^ ^150^ ^]^ and we have to come up with new concepts and ideas to fulfill the medicinal demand of radiopharmaceuticals for efficient patient care in a sustainable and environmentally friendly way. The potential impact of these aspects of radiopharmaceuticals on humans remains speculative due to the lack of available data (**Figure** **3**).

## 4 Designing Contrast Agents for a Sustainable Future

### 4.1 Guideline to Sustainable Design on Imaging Agents: Control, Change, and Combine

To countervail the increasing concerns on accumulation of medical imaging agents in the environment, here we propose the first guidelines for the design and handling of contrast/imaging agents, the 3Cs:

1. Control: controlling and preventing the release of harmful components in the environment.
2. Change: develop and implement the use of imaging agents with improved sustainability, controlled biodegradation pathways and lower toxicity.
3. Combine: enable efficient use of contrast agents by developing multipurpose agents and reduce their amounts by better combining and processing information.

Realizing these measures requires a comprehensive set of actions and inclusion of different stakeholder groups, particularly raising awareness, fostering open discussion, lobbying and facilitating communication on measures’ feasibility, while balancing interests across stakeholders. The measures are discussed in Section 4.2 and the role of stakeholders and potential challenges in Section 4.3.

#### 4.1.1 Pathway to implementing the 3C

In the light of the potential environmental impact of the increasing application of imaging agents, it is imperative to raise awareness. Our priority lies in fostering an open discussion to explore potential solutions while carefully balancing patients' rights to efficient medical treatment, the associated costs, and feasibility considerations. In this context, we aim to present various initial strategies that incorporate the principles of the 3Cs to enhance the environmental footprint of imaging agents.

#### 4.1.2 Control: Controlling and Preventing the Release of Harmful Components in the Environment

Controlling the release into the environment of existing contrast agents can primarily be realized by the collection of hospital waste, such as unused agents, which further opens an opportunity for recycling, as has been already done in the recycling programs for iodinated contrast media run, e.g., by GE Healthcare, and Bayer^[^ ^151^ ^]^ and for the controlled disposal procedures of radioactive waste in nuclear medicine.^[^ ^138^, ^142^ ^]^

To prevent the release of contrast agents into the environment after excreting from the patient, collecting patient urine can be a possible solution, and the established wastewater treatment in nuclear medicine can serve as an example. Two trials utilizing urine bags recently took place in the Netherlands and Germany, confirming a decrease of iodinated contrast media in wastewater and demonstrating general acceptance by the patients.^[^ ^152^, ^153^ ^]^ The reduced amount of iodinated contrast agents in the wastewater is promising. However, urine bags will create additional workload for hospital staff and are challenging to implement in daily hospital routine, which can become a critical point considering the current staff shortages.^[^ ^154^ ^]^ The usage of the bags will further increase the amount of special medicinal waste, which is typically incinerated and may require storage at hazardous waste landfills afterwards when they contain heavy metals such as Gd.

Improving water filtration and wastewater treatment could become an alternative here that does not require any involvement of hospital staff. These steps would require the development of membranes and other processes that can effectively remove imaging agents in WWTPs and can go hand in hand with the ongoing development for radioactive wastewater.^[^ ^155^, ^156^ ^]^ Other advanced concepts for sewage and wastewater treatment, such as combining different treatment methods, can further help to enhance the removal of imaging agents.^[^ ^121^ ^]^ However, these measures are costly and require the development of novel efficient technology.

The quickly growing demand for radiopharmaceutical theranostics is reshaping the nuclear medicine market. This trend will lead to an expansion in the applied radionuclides,^[^ ^157^ ^]^ production possibilities,^[^ ^158^ ^]^ and the required radiation waste handling procedures. This can be a driving force for academia and industry to work together on efficient solutions for the development and technology but will require high costs to implement.

Designing imaging agents with a focus on facilitating their removal in wastewater at the endpoint could offer another solution to address the challenges posed by contaminated water bodies early on. This proposed “design for removal” approach draws parallels with existing concepts such as “design for recycling” in material sciences and necessitates training early‐phase scientists to adopt a new approach in developing imaging agents.

#### 4.1.3 Change: Develop and Implement the Use of Imaging Agents with Improved Sustainability, Controlled Biodegradation Pathways, and Lower Toxicity

Change is the proactive step by changing to non‐toxic, environmentally‐friendly imaging agents. This step obviously involves the development, industrial production, and clinical approval of such agents.

Particularly, in MRI there are several promising developments, which were initially fueled by the discovery that toxic Gd‐ions can accumulate in brain and not by environmental concerns. A comprehensive development is undertaken on a few fronts ranging from different Gd‐chelates complexes to SPIOs.^[^ ^159^ ^]^ One set of promising contrast agents that can be direct replacements of Gd are manganese‐based contrast agents.^[^ ^20^, ^21^ ^]^ Despite resulting in the release of potentially toxic Mn^2+^ ions, its typical exposure upon intravenous injection results in a level below threshold of human Mn tolerability.^[^ ^160^ ^]^ This can be mitigated by the use of appropriate chelating agents, which are still actively researched,^[^ ^161^ ^]^ being comparable at clinical field strengths,^[^ ^162^ ^]^ but not yet having replaced GBCAs. However, comprehensive data on environmental and ecotoxicological effects of Mn‐chelates is still missing.

As alternative to contrasted MRI, heteronuclear MRI has shown several promising developments in the last few years. Particularly to mention is hyperpolarized MRI, mainly using dynamic nuclear polarization (DNP).^[^ ^163^ ^]^ This method allows for the fast imaging of an agent, usually [^13^C]‐Pyruvate, which metabolizes into lactate (highlighting glycolytic tissues), providing relevant metabolic information, which has found its use in human studies of prostate^[^ ^164^ ^]^ or heart.^[^ ^165^ ^]^ Despite promising results, these methods requiring separate instrumentation and a high consumption of liquid helium (this is performed typically at 1–2 K) hinders their translation and impacts their sustainability, mostly due to the latter's connection to petroleum extraction.^[^ ^166^ ^]^ A more affordable option has gained traction lately, based on deuterated glucose, leading to background‐free MRI.^[^ ^167^, ^168^ ^]^ This method is more affordable and associated with low toxicity, providing reported advantages in regards to hyperpolarized pyruvate,^[^ ^169^ ^]^ which already made it a leading method in human studies.

Moreover, several potential alternatives to PFAS have been introduced in the last few years. Specifically, inorganic nanofluorides,^[^ ^170^, ^171^ ^]^ based on CaF_2_ for example, allow for multimodal imaging with MRI and CT.^[^ ^172^ ^]^ Moreover, the development agents for ^31^P MRI which was previously hampered by the natural background and low sensitivity of the nucleus has increased in recent years. The recent development of ^31^P‐based polymeric imaging agents can also provide a viable alternative,^[^ ^173^ ^]^ including biodegradable polymers (polyphosphoesters) suitable for rapid imaging sequences which recently became commercial.^[^ ^174^, ^175^ ^]^ All these agents are now in preclinical studies but may pave the way to biocompatible and biodegradable imaging agents but translational efforts are now necessary to realize the leap from bench to bedside.

Also, for ultrasound imaging, replacing PFAS would help to reduce the environmental burden. Over the last decades, the role of lipid shell formulation on bubble stability has been investigated in several works, e.g., to enable oxygen delivery with O_2_ filled bubbles. Long lipid acyl chain lengths have been shows to increase the resistance of the lipid shell to diffusive gas transport.^[^ ^176^ ^]^ A similar direction may be followed to further optimize bubble stability and thereby enable the use of, for example, air as a bubble filling gas in UCAs thereby fully omitting the use of PFAS.

In CT, the high dose makes the reduction of the agent dose necessary. Various heavy metal based nanoparticles, including gold, Gd, and composite based on biodegradable polymers that encapsulate metal colloids^[^ ^177^ ^]^ and are now investigated as CT agents and could potentially lead to strongly reducing the dose of contrast media. Some of these materials, particularly gold, are considered safe and biocompatible, yet there are also examples of materials based on toxic metals, such as Gd. To our knowledge, there is no nanoparticle‐based CT agent approved for clinical use. Nevertheless, several gold formulations are in clinical trials for other applications which should foster clinical translation of gold‐based CT agents.^[^ ^178^ ^]^ One, however, should keep in mind that similarly to iodine all metals are non‐renewable, require mining, some, e.g., Gd are toxic, and can potentially lead to environmental harms upon release in the environment. Thus, while metal nanoparticles could be a solution to reduce the contrast media dose, all the other aspects of their use should also be considered by implementing combined measures which include not just lowering the dose but also collecting the waste.

In nuclear medicine, an increasing number of publications occur addressing the positive effect on the environmental impact, when changing different parameters within the highly repetitive radiopharmaceutical manufacturing process. Changing from Germanium/gallium generators to a gallium‐68 production in medical cyclotrons does not only lower the costs, but significantly reduces the carbon footprint of the required transportation of the generators around the globe.^[^ ^179^, ^180^ ^]^ However, the ongoing change was not initiated based on sustainability reasons, but mainly because of availability. Another initiative involves the change of organic solvents to greener options like ethanol or water, especially for fluorine‐18 based productions.^[^ ^181^, ^182^, ^183^, ^184^ ^]^ In general, sustainability considerations should also already be included for the choice of radionuclide applied in nuclear medicine, however, this was so far not systematically studied.

Finally, changing to a different imaging modality, which does not require using of harmful agents provides a viable alternative. Currently, optical and optoacoustic imaging are progressing and enabling the detection deeper in the tissue, and at the same time enabling super‐resolution imaging and sensing.^[^ ^185^, ^186^, ^187^, ^188^, ^189^, ^190^ ^]^ Both methods usually exploit organic molecules as fluorophores and chromophores, omitting heavy metals and radioactive waste. Currently, optical probes are only approved for the intraoperative imaging, and optoacoustic scanners entered clinics just a few years ago. The future development of both technologies for deep tissue imaging could enable broadening their applications and reducing the environmental footprint of medical imaging.

#### 4.1.4 Combine: Enable Efficient Use of Contrast Agents by Developing Multipurpose Agents and Reduce Their Amounts by Better Combining and Processing Information

Advancing technology is the driving force for the concept of combine and connect. Multimodal imaging agents, which can be detected with several modalities, are often more efficient than classical single modality agents in regard to the information that one can obtain imaging, e.g. combining quantification, with longitudinal monitoring. Combining the information from different techniques could along with the development of processing and prediction algorithms could indeed eventually reduce the intervals between imaging sessions when monitoring the disease progression or healing processes.

However, so far, the design often comes with downsides in the image quality in a single modality versus classical agents. For example, agents for multimodal imaging with ultrasound and ^19^F‐MRI, based on liquid PFAS do not hold up to microbubbles in ultrasound imaging.^[^ ^191^, ^192^, ^193^, ^194^, ^195^, ^196^ ^]^ On the other hand, they offer an increased stability which could potentially allow to reduce the dose in the case new routes in the future enable optimizing the acoustic performance. Another promising development is the design of multiscale agents which can be detected at different magnification levels, such as a superfluorinated PERFECTA molecule which produces ^19^F MRI for the detection at a whole body scale and a Raman signal at a cellular scale.^[^ ^197^, ^198^, ^199^ ^]^ This design approach could be extended in the future to the development of partly biodegradable fluorinated probes, combined with collection and ideally recycling of the fluorinated units.

Also in MRI, image contrasts based on chemical exchange saturation transfer (CEST)^[^ ^200^, ^201^ ^]^ can provide intrinsic contrast based on metabolites,^[^ ^202^, ^203^ ^]^ or on proteins (amide proton weighting, APT).^[^ ^204^, ^205^, ^206^ ^]^ These can strongly support MRI scans, diminishing the need for contrast agents. The future exploration of other forms of intrinsic and extrinsic contrast and artificial intelligence based solutions can further open avenues to a more comprehensive clinical portfolio.^[^ ^207^, ^208^, ^209^, ^210^ ^]^ Furthermore, the combination of iodinated CT agents with CEST‐based methods can lead to pH imaging^[^ ^211^ ^]^ and present a viable avenue towards multimodality.^[^ ^212^ ^]^

Nuclear medicine was pioneering on the effectiveness and strength of combining methods with PET/CT and SPECT/CT hybrid systems in routine application. In PET, total‐body scanners allow a significant reduction of applied radiotracers, thereby reducing radiochemical waste and improving the output of a medical imaging scan.^[^ ^213^ ^]^

Approaches including artificial intelligence and machine learning will contribute to denoising and removal of artifacts of ultra‐low dose imaging. Efficient attenuation correction and image reconstruction has the potential to improve the readout of imaging results.^[^ ^214^ ^]^ Additionally, the strides made by deep learning and artificial intelligence methods in radiology^[^ ^213^ ^]^ can help minimize the amount of contrast agents required, or overall scanning times, which can lead to a positive environmental impact, though it is still a developing subject.^[^ ^215^ ^]^

### 4.2 The Role of Stakeholders

**Figure 4:**
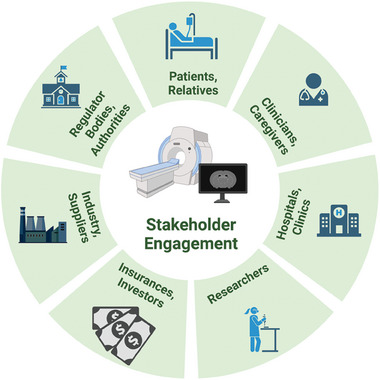
Stakeholders involved in the process of creating sustainable solution for medical imaging and the realization of the 3Cs for imaging agents. Each stakeholder will have different value prioritization that have to carefully be balanced.

A practical and sustainable solution can only be realized when the interests of all involved stakeholders are identified. Identified stakeholders include the clinical, academic, industrial, and legal/regulatory sectors (**Figure** **4**). Within the clinical sector, patients, clinicians, caregivers, and hospital administration will have to work together and understand these different viewpoints to come up with practical solutions. To achieve the solution, each of the above groups play a particular role:

• The hospital administration holds the responsibility to introduce the measures, while caregivers and clinicians must support them by applying and explaining the new solutions to the patients and patient organizations. Patient awareness must be raised to ensure compliance with the measures, such as actively collecting urine.
• The academic sector has the potential to develop imaging agents with reduced environmental impact, which can then be produced by the industry and supply producers, leading to, e.g., approaches to limit plastic consumables or improved wastewater treatments. A consolidation of the clinical, academic, and industry sectors can be promoted, carried out, and strengthened by regulatory bodies that establish frameworks (e.g., public guidelines) for future regulatory acts, implement policies, and potentially provide funding for interdisciplinary research.
• Insurance companies (or other organization responsible for financing health) need to balance financial interests and providing solutions for affordable care. Practices for economic sustainability may include developing consolidated guidelines and strategic coordination, that align financial and sustainable initiatives, and communicating these to all stakeholders.
• Policymakers and regulatory organizations, such as EMA, FDA, environmental ministries can facilitate the transfer of sustainable agents to the market by for example adjusting the clinical approval regulations and creating new testing guidelines which include for example defined protocols for degradation testing for biodegradable agents. Additionally, implementing the requirement for environmental safety and ecotoxicology testing should ensure that the agents are tested sufficiently and omit environmental issues. Finally, limiting the use of for example persistent agents can further increase the market uptake of newer formulations which may otherwise struggle entering a strongly consolidated contrast agent market.
• Universities and other educational organizations play a key role in incorporating sustainability aspects into established training programs for medical imaging in secondary and tertiary education will not only convey the importance of sustainability to researchers but also to industry and regulatory bodies, as these students will become future employees and help ensure long‐term solutions. A circular educational approach should include external lecturers from industry, regulatory bodies, and clinics within the training programs to diversify the topic.

### 4.3 Opportunities and Challenges

There are both opportunities and challenges involved in the transition from the current approach to an equally successful, financially affordable and additionally sustainable medical imaging.

Addressing the specific structures in a particular region in hospitals is essential to aligning the measures within the stakeholders’ groups. As a starting point here, we suggest raising awareness by incorporating sustainability topics into established international meetings and discussion forums. This will kick off the discussion between imaging and environmental experts, which can help to later involve other relevant stakeholders at national and local levels. Strategy development must be guided by strong coordination and collaboration with scientific boards, and focus groups that involve all relevant stakeholders. At the early stage, inter‐ and cross disciplinary project that allow for involvement of all stakeholders can pave the way to an in‐depth discussion. Later, a more specific measures can be implemented at the national level considering country‐dependent specifics, eventually improving sustainability.

There are several barriers to the development and implementation of sustainable imaging agents, including the lack of data, guidelines, solutions, and high costs. The market for contrast agents is quite consolidated and dominated by big players but still offers some space for niche products that can sometimes even create new markets, as happened for radiopharmaceutical theranostics in the past few years. Medical imaging agents are classified as medicinal substances or medical imaging drugs by EMA and FDA, meaning that they have to follow the clinical trial route. The high costs can hamper the market entry. Adjustment of requirements for clinical approval and standardizing the process for, e.g., biodegradable agents, can facilitate the market entry. Moreover, implementing measures for more efficient knowledge transfer from academic institutions to start‐ups and industry can further support the market entry of new environment‐friendly solutions.

The limited and heterogeneous scientific evidence and lack of information on the long‐term effects of medical imaging agent exposure in the environment further affect not only the establishment of stakeholder engagement, educational competencies, and regulatory frameworks. Here, a close collaboration between different sectors can offer numerous opportunities to strengthen policy across academia, healthcare, community organizations, and policymakers. This solution‐oriented collaboration has the potential to foster innovation for both efficient and sustainable medical imaging, advancing technical and economic progress. Incorporating sustainability thinking early in educational and training programs will help build diverse knowledge and prepare students for the challenges of balancing medical imaging with environmental impact.

In overall, understanding the involved different viewpoints and implementing a platform to effectively communicate with the stakeholders will help to work together on creating scientific evidence and sustainable solutions in a more efficient and collaborative manner and the well‐being of patients.

## 5 Conclusion

Medical imaging is important to save lives and to initiate efficient treatment of many diseases. However, the widespread use of current contrast agents in medical imaging results in their environmental accumulation, posing risks to human health and the environment. Today's contrast agents mostly rely on potentially toxic heavy metals or non‐biodegradable substances: Gadolinium‐based agents, PFAS/PFCs, and iodinated agents have been detected in several waters. Radioactive decay products from radiopharmaceuticals also requires careful management. To address these issues, urgent efforts are needed to develop and adopt environmentally friendly and biodegradable alternatives, such as biodegradable polymers or natural pigments. Embracing these alternatives will enable the continued benefits of medical imaging while minimizing adverse impacts on the environment. The 3C strategy proposed here can become a first step to reduce the pollution by medical imaging agents, yet it requires further discussion and actions by different stakeholders, including clinicians, hospital managers, insurances, governments, and patients.

## Conflict of Interest

The authors declare no conflict of interest.
